# Supplementary material for: HIVprotI: an integrated web based platform for prediction and design of HIV proteins inhibitors
Source: J Cheminform. 2018 Mar 9;10:12. doi: 10.1186/s13321-018-0266-y (PMC5845081; doi:10.1186/s13321-018-0266-y)
Supplement: Supplementary file 2 — Additional file 2. Source code of HIVProtI web server. [file 13321_2018_266_MOESM2_ESM.zip › HIVprotI_Source-code/hivprotI/weka-3-6-6/doc/weka/core/pmml/package-summary.html]

weka.core.pmml


---


|  |  |  |
| --- | --- | --- |
| **Overview** \| **Package** \| Class \| **Tree** \| **Deprecated** \| **Index** \| **Help** \| **Weka's home** | |  |
| **PREV PACKAGE**   **NEXT PACKAGE** | **FRAMES**    **NO FRAMES**     **All Classes** |  |


---

## Package weka.core.pmml

| **Interface Summary** | |
| --- | --- |
| **PMMLModel** | Interface for all PMML models |

| **Class Summary** | |
| --- | --- |
| **BuiltInArithmetic** | Built-in function for +, -, \*, /. |
| **BuiltInMath** | Built-in function for min, max, sum, avg, log10, ln, sqrt, abs, exp, pow, threshold, floor, ceil and round. |
| **BuiltInString** | Built-in function for uppercase, substring and trimblanks. |
| **Constant** | Class encapsulating a Constant Expression. |
| **DefineFunction** | Class encapsulating DefineFunction (used in TransformationDictionary). |
| **DerivedFieldMetaInfo** |  |
| **Discretize** | Class encapsulating a Discretize Expression. |
| **Expression** |  |
| **FieldMetaInfo** | Abstract superclass for various types of field meta data. |
| **FieldMetaInfo.Interval** | Inner class for an Interval. |
| **FieldMetaInfo.Value** | Inner class for Values |
| **FieldRef** | Class encapsulating a FieldRef Expression. |
| **Function** | Abstract superclass for PMML built-in and DefineFunctions. |
| **MappingInfo** | Class that maintains the mapping between incoming data set structure and that of the mining schema. |
| **MiningFieldMetaInfo** | Class encapsulating information about a MiningField. |
| **MiningSchema** | This class encapsulates the mining schema from a PMML xml file. |
| **NormContinuous** | Class encapsulating a NormContinuous Expression. |
| **NormDiscrete** | Class encapsulating a NormDiscrete Expression. |
| **PMMLFactory** | This class is a factory class for reading/writing PMML models |
| **PMMLUtils** | Utility routines. |
| **TargetMetaInfo** | Class to encapsulate information about a Target. |

| **Enum Summary** | |
| --- | --- |
| **FieldMetaInfo.Interval.Closure** | Enumerated type for the closure. |
| **FieldMetaInfo.Optype** | Enumerated type for the Optype |
| **FieldMetaInfo.Value.Property** | Enumerated type for the property. |

---


|  |  |  |
| --- | --- | --- |
| **Overview** \| **Package** \| Class \| **Tree** \| **Deprecated** \| **Index** \| **Help** \| **Weka's home** | |  |
| **PREV PACKAGE**   **NEXT PACKAGE** | **FRAMES**    **NO FRAMES**     **All Classes** |  |


---
